# Coach-Facilitated Web-Based Therapy Compared With Information About Web-Based Resources in Patients Referred to Secondary Mental Health Care for Depression: Randomized Controlled Trial

**DOI:** 10.2196/15001

**Published:** 2020-06-09

**Authors:** Sarah MacLean, Daniel J Corsi, Sadie Litchfield, Julia Kucharski, Kira Genise, Zeynep Selaman, Valerie Testa, Simon Hatcher

**Affiliations:** 1 School of Journalism and Communication Carleton University Ottawa, ON Canada; 2 Clinical Epidemiology Program Ottawa Hospital Research Institute Ottawa, ON Canada; 3 Children's Hospital of Eastern Ontario Research Institute Ottawa, ON Canada; 4 Better Outcomes Registry & Network Ontario Children's Hospital of Eastern Ontario Ottawa, ON Canada; 5 School of Epidemiology and Public Health University of Ottawa Ottawa, ON Canada; 6 Prevention and Rehabilitation University of Ottawa Heart Institute Ottawa, ON Canada; 7 Department of Psychiatry The Ottawa Hospital Ottawa, ON Canada; 8 Faculty of Medicine University of Ottawa Ottawa, ON Canada; 9 Department of Psychiatry University of Ottawa Ottawa, ON Canada; 10 Interdisciplinary School of Health Sciences University of Ottawa Ottawa, ON Canada

**Keywords:** major depressive disorder, secondary care, randomized controlled trial, telemedicine, digital health technologies, Canada

## Abstract

**Background:**

Depression is a common mental disorder with a high social burden and significant impact on suicidality and quality of life. Treatment is often limited to drug therapies because of long waiting times to see psychological therapists face to face, despite several guidelines recommending that psychological treatments should be first-line interventions for mild to moderate depression.

**Objective:**

We aimed to evaluate, among patients on a waitlist to receive secondary mental health care services for depression, how effective coach-guided web-based therapy (*The Journal*) is, compared with an information-only waitlist control group, in reducing depression symptoms after 12 weeks.

**Methods:**

We conducted a randomized controlled trial with 2 parallel arms and a process evaluation, which included interviews with study participants. Participants assigned to the intervention group received 12 weeks of web-based therapy guided by a coach who had a background in social work. Patients in the control group receive a leaflet of mental health resources they could access. The primary outcome measure was a change in depression scores, as measured by the Patient-Health Questionnaire (PHQ-9).

**Results:**

A total of 95 participants were enrolled (intervention, n=47; control, n=48). The mean change in PHQ-9 scores from baseline to week 12 was −3.6 (SD 6.6) in the intervention group and −3.1 (SD 6.2) in the control group, which was not a statistically significant difference with a two-sided alpha of .05 (t_91_=−0.37; *P*=.72, 95% CI −3.1 to 2.2). At 12 weeks, participants in the intervention group reported higher health-related quality of life (mean EuroQol 5 dimensions visual analogue scale [EQ-5D-VAS] score 66.8, SD 18.0) compared with the control group (mean EQ-5D VAS score 55.9, SD 19.2; t_84_=−2.73; *P*=.01). There were no statistically significant differences between the two groups in health service use following their initial consultation with a psychiatrist. The process evaluation showed that participants in the intervention group completed a mean of 5.0 (SD 2.3) lessons in *The Journal* and 8.8 (SD 3.1) sessions with the coach. Most participants (29/47, 62%) in the intervention group who completed the full dose of the intervention, by finishing 6 or more lessons in *The Journal*, were more likely to have a clinically important reduction in depressive symptoms at 12 weeks compared with the control group (Χ^2^_1_=6.3; *P*=.01, Φ=0.37). Participants who completed the interviews reported that the role played by the coach was a major factor in adherence to the study intervention.

**Conclusions:**

The results demonstrate that the use of guided web-based therapy for the treatment of depression is not more effective than information-only waitlist control. However, it showed that the coach has the potential to increase adherence and engagement with web-based depression treatment protocols. Further research is needed on what makes the coach effective.

**Trial Registration:**

ClinicalTrials.gov: NCT02423733; https://clinicaltrials.gov/ct2/show/NCT02423733

## Introduction

### Background

Depression is a common mental disorder [1] with a high social burden [2] and significant impact on suicidality [3] and quality of life. Most treatment for depression occurs in primary care. In secondary care, treatment is often limited to drug therapies, in part, due to long waiting lists to see psychological therapists face-to-face. This is contrary to recommendations about the importance of nondrug therapies by the National Institute for Health and Care Excellence (NICE), based in the United Kingdom, and other institutions [4]. In secondary and tertiary mental health care centers in Ontario, at the time of this trial, the waiting time to be seen by a psychiatrist for depression was between 9 months and 1 year.

### Use of Electronic Therapies in Treating Depression

There is evidence that web-based therapies can reduce the symptoms of depression [5]. Randomized controlled trials (RCTs) have demonstrated the effectiveness of web-based cognitive behavior therapy (CBT) [6,7], problem-solving therapy [8], interpersonal therapy [9], and psychodynamic therapy. Computerized CBT is also recommended in the NICE guidelines for the treatment of mild to moderate depression [4]. However, most of the RCTs of web-based therapies have been conducted in community samples, often recruited from the internet. These populations are self-selecting and, although their scores on depression rating scales may be comparable with clinical populations, they often differ in terms of comorbidity, duration of symptoms, and impact on daily functioning.

### Guided Electronic Therapy in the Treatment of Depression

The delivery of web-based therapy as a treatment for depression can be performed in one of two ways: through the use of supports to assist patients through the web-based therapy (guided model) or through the self-help use of computerized treatment (unguided model). In the guided model, patients are provided support as they progress through web-based therapy. In some cases, highly trained clinicians have been used to fill this role [10]; however, their advanced training is costly and does not appear to provide any added benefit. For instance, despite the use of a clinician in the delivery of web-based therapy for the treatment of depression and anxiety among young adults, Dear et al [11] did not find any significant differences in symptomatology when compared with the unguided condition. One solution that has been implemented to mitigate this issue is the use of coaches to support patients progressing through web-based therapy, answering both technical questions about how the program works as well as providing support and encouragement. To date, coaches have included both students [12] and licensed professionals from a variety of backgrounds, including psychology [13], counseling [14], and social work [15,16]. The components of effective coaching are still uncertain [17], with most of the literature emphasizing the technical aspects of an internet intervention. It is not clear whether the professional background of the coach, the frequency of contact, or the content of the coaching sessions affects treatment outcomes.

Several systematic reviews have demonstrated that internet-based interventions for depression have effect sizes that are comparable with face-to-face interventions, whereas unguided interventions have smaller effect sizes [7,18,19]. However, the evidence is inconsistent, with head-to-head comparisons of guided versus unguided interventions showing mixed results. The largest study of guided web-based therapy compared with usual care has been the Randomised Evaluation of the Effectiveness and Acceptability of Computerised Therapy (REEACT) study [20], which was a UK-based RCT of 691 patients with depression in primary care. Participants were randomized to one of three treatment groups: (1) usual care; (2) *Beating the Blues*, a guided commercial web-based program; or (3) *MoodGYM*, a guided free web-based program. The study found no difference in depression outcomes between the three groups. Similarly, Kenter et al [21] compared student-assisted internet-based problem-solving therapy with an information-only waitlist control and found minimal differences in depression severity (Cohen *d*=0.07), as measured by the Centre for Epidemiology Studies Depression scale.

### Adherence to Web-Based Therapy Treatment Protocols

Web-based interventions for depression struggle with engagement and adherence to treatment protocols. For example, in the *Beating the Blues* and *MoodGYM* arms of the REEACT trial, participants completed only a median of 1 to 2 sessions and received only 6 min of technical support time, 5 emails, and almost no text messages from the telephone support workers. Further, approximately 1 in 5 participants randomized to either of the web-based therapy conditions did not access the programs at all. Similar problems were encountered in the study conducted by Kenter et al [21], with only 36% of participants receiving an adequate dosage of the intervention, defined by the authors as 4 of 5 lessons of web-based therapy. From a public health perspective, low adherence to web-based therapies dilutes their effectiveness. However, it is unclear if, for individual patients, adhering to a complete course of web-based therapy is better than control treatments.

### This Study

We have previously reported on an RCT of *The Journal* comparing guided web-based therapy with an information-only waitlist control in patients with depression referred to secondary mental health services in New Zealand [22]. Participants were recruited face-to-face during their triage visit at community mental health centers, and the study found no difference in depression or service use after 12 weeks. However, all participants in this study were also receiving mental health care from clinicians, which may have outweighed any effect of the web-based therapy. In this study, we report on an RCT of *The Journal* [23]*,* facilitated by a coach, compared with an information-only waitlist control group for the treatment of depression in patients referred to secondary mental health services in Canada. In this setting, access is a significant issue, with patients often waiting for over a year to access mental health services. During this time, patients on the waiting list do not receive any other mental health care other than routine follow-up from their family physician; therefore, the provision of web-based therapy could potentially be used as an alternative to referral to specialized mental health services. This study gave us an opportunity to refine the study intervention. In contrast to the New Zealand study, we opted to use an information-only control group as it is a low-cost, low-risk alternative that could easily be implemented in clinical practice. Similarly, we were able to refine the coaching aspect of the intervention. We hypothesized that, after 12 weeks of treatment, participants receiving coach-guided web-based therapy would experience a greater reduction in depressive symptoms and health service use.

## Methods

### Trial Design

The design of the trial was an RCT with two parallel groups. This trial has been reported according to the Consolidated Standards of Reporting Trials of Electronic and Mobile Health Applications and Online Telehealth (CONSORT-EHEALTH; Multimedia Appendix 1).

### Recruitment

Potential participants were patients referred to the Royal Ottawa Mental Health Centre (ROMHC, Ottawa, Canada) with symptoms of depression or dysthymia who were on a waiting list for treatment in the following psychiatric programs: Mood and Anxiety, Geriatric Psychiatry and Youth Psychiatry. The ROMHC has 284 inpatient beds and acts as a specialized mental health facility for residents of communities across Eastern Ontario. These programs are aimed at treating people with complex and serious mental illnesses that are often resistant to treatment. Patients are referred to treatment in these programs directly by their family physicians. The first appointment in the programs is with a psychiatrist who then decides on a treatment plan with the patient. At the time of this clinical trial, the waitlist for the Mood and Anxiety Program was between 9 months and 1 year.

Eligibility criteria for participation in the trial are outlined in Textbox 1. Potentially eligible patients were contacted using the following methods: patients who had completed a consent to be contacted for research as part of their referral documentation were contacted by telephone. Those who did not complete the consent to be contacted for the research section of the referral were contacted by mail, as per institutional policies. Interested patients were preliminarily screened by a research assistant for eligibility to participate in the study. Eligible patients were then asked to attend a face-to-face consenting appointment with a research assistant. A minority of patients who could not travel to the clinic were asked to consent via mail (n=3).

**Participant eligibility criteria.**

Inclusion criteria:

- 16 years of age or older.
- Referred and triaged to the Mood and Anxiety, Youth Psychiatry, or Geriatric Psychiatry Program at the Royal Ottawa Mental Health Centre for any depressive symptoms.
- Willing to attend electronic therapy sessions for up to 12 weeks.
- Able and willing to provide informed consent.
- Willing to be randomized.
- Willing to comply with all study procedures.

Exclusion criteria:

- Is unable to read or write in English.
- Does not have an Ontario Health Insurance Plan number.
- Has cognitive impairments that render them unable to use a computer.
- There is another participant enrolled in the study who lives at their address.

### Interventions

#### The Journal

*The Journal* [23] is an evidence-based free web-based therapy program developed in New Zealand for the self-management of depression that utilizes the cognitive behavioral techniques of behavioral activation and problem solving (Multimedia Appendix 2). The problem-solving approach was derived from a large RCT of face-to-face problem-solving used in people who presented to emergency departments with intentional self-harm [24].

As described in Table 1, there are a total of 9 modules in *The Journal*, 6 of which must be done for patients to complete the program. Participants progressed through the web-based therapy as follows: (1) positivity module (1 lesson); (2) lifestyle modules (4 lessons, 1 of which must be completed); and (3) problem-solving module (4 lessons). At the start of each lesson, users are asked to watch a video featuring Sir John Kirwan, a former All-Blacks rugby player, who has been very public about his own struggles with depression in an effort to reduce stigmatization.

**Table 1:** Breakdown of participant progress through The Journal.

Lessons			Description		Tasks to be completed by participants
Positivity module			Learn the importance of staying positive and planning regular activities that they enjoy		Watch video on staying positive; Select 2 enjoyable activities; Select dates to complete activities.
**Lifestyle module^a^**					
	Eating right	Explore the link between diet and mood.		Watch video on eating right; Browse and select a healthy recipe; Create a shopping plan.	
	Getting active	Review benefits of being active on mood.		Watch video on getting active; Pick 2 activities to complete; Make a plan for getting active.	
	Learning to relax	Highlights the importance of stress management.		Watch video on learning to relax; Practice relaxation and breathing; Make a plan for relaxing exercises.	
	Sleeping better	Discuss the importance of good sleep habits to mood.		Watch video on sleeping better; Set a nighttime routine; Set a morning routine; Keep a sleep diary; Make a plan to practice sleep hygiene.	
**Problem-solving module**					
	Identify problems	Learn how depression impacts problem-solving abilities.		Watch video on identifying problems; Create a problem list; Pick a problem to work on; Define the problem; Make a plan to create a problem list and statement.	
	Find solutions	Explore how to use both logical and creative parts of the brain to brainstorm problem solutions.		Watch video on brainstorming solutions; Create a solutions list; Select a solution to implement; Evaluate solutions; Make a plan to list and evaluate solutions.	
	Create a plan	Review how to create SMART^b^ plan.		Watch video on brainstorming solutions; Review the selected solution to make sure it is SMART; Write a detailed step-by-step plan; Review plan.	
	Review your plan	Highlights the importance of assessing progress and updating the SMART plan.		Watch video on reviewing the SMART plan; Review progress on plan; Revise plan as needed; Complete self-test.	

^a^Participants are only required to complete 1 of the 4 lifestyle lessons.

^b^SMART: specific, measurable, achievable, relevant, time-bound.

#### Study Intervention

Both groups received usual care while on the waitlist, which included management by a family physician and use of community resources, such as access to distress center lines and counseling services. Once participants are called off the waitlist, they receive an initial appointment with a psychiatrist, at which point adjustments are made to the patient’s care plan. They are then referred back to their family physician, or they receive further psychiatric treatment (eg, regular care from a psychiatrist, social worker, nurse, occupational therapist, and so on).

Following the consent appointment, participants were randomized to one of two treatment groups. In addition to usual care, participants assigned to the control group received an information leaflet with web-based resources, including *The Journal*, and told that they could decide for themselves the best way to use this information while on the waitlist. Participants had been previously informed by their clinical team that the estimated wait time to be seen by a psychiatrist was between 9 and 12 months.

For those participants assigned to the intervention group, the intervention consisted of the following:

1. An information leaflet of web-based depression resources.
2. An invitation to use *The Journal.*
3. 12-weekly telephone coaching sessions with a coach (SL), who had a guideline script for each coaching session, reinforced the topic of each lesson in *The Journal*, helped identify and support participants in goal setting and the techniques of problem-solving. Each session lasted between 30 and 60 min. The coach had a background in social work and received weekly supervision from the principal investigator (SH).
4. Text message or email contact between appointments, as per the participant’s preference.

### Outcomes

#### Primary Outcome

The primary outcome measure was the Patient Health Questionnaire (PHQ-9) [25], a 9-item questionnaire that assesses the severity of depression symptoms experienced within the preceding 2 weeks. Participants are asked to rate each symptom of depression on a Likert scale from 0 (not at all) to 3 (nearly every day), with total scores ranging from 0 (minimal depression) to 27 (severe depression). The PHQ-9 has strong methodological properties, with an internal consistency of 0.89 and strong test-retest reliability [26]. Increasing scores on the PHQ-9 have also been found to be correlated with deteriorating scores on all 6 subscales of the Medical Outcomes Survey Short Form-20 [27]. The PHQ-9 was selected not only for its strong psychometric properties but also for its commonality. The PHQ-9 is often used as a screening tool for major depressive disorder in primary care practice [28].

#### Secondary Outcomes

Suicidal thoughts were assessed by question 9 of the PHQ-9, in which respondents were asked *Over the last 2 weeks how often have you been bothered by thoughts that you would be better off dead or of hurting yourself in some way*? [26]. This variable was dichotomized as follows: participants who responded *Not at All* (0) were categorized as *no* (0), and participants who reported any degree of suicidality (1, 2, or 3) were categorized as *yes* (1).

Health-related quality of life was assessed using the EuroQol-5 dimension (3 levels) questionnaire (EQ-5D-3L). This is a 5-item questionnaire that assesses health-related quality of life, including mobility, self-care, ability to participate in one’s usual activities, pain or discomfort, and anxiety or depression. The EQ-5D-3L asks participants to assess their health-related quality of life on a 3-point scale from no dysfunction to extreme dysfunction, with the following response categories:

- Level 1: indicating no problem.
- Level 2: indicating some problems.
- Level 3: indicating extreme problems.

The EQ-5D-3L is then able to define a unique health state based on the responses to each of the 5 dimensions of health described above. Respondents fall into 1 of 243 different health states, depending on their responses to the questionnaire [28]. For instance, an overall score of 11111 indicates no problems in any of the 5 health dimensions, whereas a score of 12312 indicates that a respondent has no problems with mobility, some problems with washing or dressing, extreme problems with doing usual activities, no pain or discomfort, and some anxiety or depression. The measure also includes a visual analogue scale (VAS), which asks participants to evaluate their overall health on a scale from 0 to 100. The EQ-5D-3L has strong psychometric properties and has been found to be moderately to highly correlated with other measures of impairment and disability [29,30].

Service use was measured using data extracted from participants’ electronic medical records (EMR), including, time to first consultation appointment at the ROMHC and the total number of outpatient mental health follow-up appointments after the first consultation completed at the ROMHC. These measures were administered as shown in Table 2.

Baseline assessments were administered in-person following the consent appointment, and all other time point assessments were conducted by telephone either by the coach (intervention group) or a research assistant (control group). Patients who missed appointments or were lost to follow-up were also sent questionnaires by mail. Service use at the ROMHC was obtained from the EMR of each participant. Five participants in the control group were excluded ad hoc to prevent confounding as they accessed *The Journal* during the treatment period.

**Table 2:** Outcome measures and timing of assessments.

Variable		Outcome measure	Time point
**Primary outcome**			
	Depressive symptoms	PHQ-9^a^	Baseline, week 2, week 6, week 12
**Secondary outcomes**			
	Suicidal thoughts	PHQ-9 Q9^b^	Baseline, week 2, week 6, week 12
	Health-related quality of life	EQ-5D-3L^c^, EQ-5D-VAS^d^	Baseline, week 6, week 12
	Health service use	ROMHC EMR^e^	One year following the initial consultation appointment at the ROMHC

^a^PHQ-9: Patient Health Questionnaire.

^b^PHQ-9 Q9: Patient Health Questionnaire question 9.

^c^EQ-5D-3L: EuroQol 5 dimensions (3 levels) questionnaire.

^d^EQ-5D-VAS: EuroQol 5 dimensions visual analogue scale.

^e^ROMHC EMR: Royal Ottawa Mental Health Center electronic medical record.

### Sample Size

Based on the previous studies that used the PHQ-9 as their primary outcome measure, we expected the mean pretreatment score to be 17.0 (SD 4.0). To detect a difference in PHQ-9 scores between the two groups of at least 3 points, an established minimal clinically important difference [31], we would need 44 participants in each group with a 2-sided alpha of .05, and a power of 80.0% (effect size of 0.6). Allowing for a 25.0% dropout rate, we aimed to recruit a total of 110 participants.

### Randomization

Randomization was completed by the Ottawa Methods Centre at the Ottawa Hospital Research Institute, with allocations kept in sequential sealed envelopes at the study base. Participants were randomized in a 1:1 allocation, and there were no restrictions. After providing consent, participants were randomized by a research assistant according to the allocation in the sealed envelopes.

### Blinding

Owing to the nature of the intervention, neither participants nor study staff were blinded to the treatment allocation. Outcome assessments were collected by delegated study staff who were not blinded to the treatment allocation.

### Statistical Analyses

Group differences in demographic and pretreatment measures were analyzed using independent samples *t* tests. Changes in participants’ scores from pretreatment to follow-up at 12 weeks were assessed using repeated measures analysis of variance with mixed linear modeling to account for missing variables. This model included the following variables: treatment group (control or intervention), PHQ-9 scores at 4 different time points (baseline, week 2, week 6, and week 12), gender (male or female), and age. PHQ-9 scores were entered as within-subject variables, treatment group as between-subject factors, and age and gender as covariates. Statistical analyses were conducted using the general linear modeling repeated measures procedure in IBM SPSS Statistics 25 for Windows. To assess significant differences in outcome measures, the last observations for the PHQ-9, EQ-5D-3L and EQ-5D-VAS were carried forward for the 6- and 12-week time points.

Differences in suicidal thoughts between the two groups were assessed using an independent samples *t* test. Differences in proportions of service use were assessed using chi-square tests. In addition, we also assessed whether participants experienced a clinically important reduction in depression symptoms defined as a PHQ-9 score of 9 or less or a 50.0% reduction in scores [31]. This was described using percentages and frequencies, and chi-square was used to assess the significance of the differences in proportions. Assessments of normality were completed using the Shapiro-Wilk test for continuous data, and the Mann-Whitney U test for nonparametric data.

### Process Evaluation

As per the recommendations outlined in the Medical Research Council’s guidelines for the assessment of complex interventions [32], we conducted a process evaluation to assess the context, implementation, and mechanisms of impact of the study intervention. The process evaluation outcome measures are outlined in Table 3. We also conducted semistructured interviews with participants within 6 months of study completion to assess their experience of the study intervention, including access to and functionality of *The Journal*, the therapeutic content and value of *The Journal*, and the experience of working with a coach. Interviewees were identified using a purposive sampling approach and were stratified to include participants in both arms of the trial and varying levels of engagement (eg, not engaged, moderately engaged, and highly engaged). Interviews were conducted until data saturation was reached (n=15).

**Table 3:** Process evaluation outcome measures.

Evaluation critierion and outcome measure		Description	
**Context**			
	Facilitators of and barriers to study completion		Qualitative interviews with participants
**Implementation**			
	Reach		Total number of participants reached Comparison of sample to Ontarian and Canadian populations.
	Fidelity		Mean length of weekly coaching calls Mean number of contacts with the coach Mean number of weekly coaching sessions completed Mean number of lessons in *The Journal*^a^
	Dose		Total number of participants to complete 6 lessons in *The Journal*
**Mechanisms of impact**			
	Role of the coach		Qualitative interviews with participants

^a^All usage data from *The Journal* were assessed via participant self-report.

Individual interviews were conducted by two female University of Ottawa Department of Psychiatry residents (JK and ZS) who were independent of the research team that conducted the RCT. Following transcription, two independent coders (JK and KG) analyzed the material using a thematic, grounded theory approach. The coding took place in two stages, with coders meeting during the first stage to discuss emergent codes, reconcile definitions, and compare coding rationales. The two coders had not been involved in delivering the treatment or any of the previous study contacts.

### Ethics

The study received approval from the Royal Research Ethics Board (Protocol 2014001). All participants provided informed consent before participation in both the RCT and qualitative interviews.

## Results

### Participants

**Figure 1:**
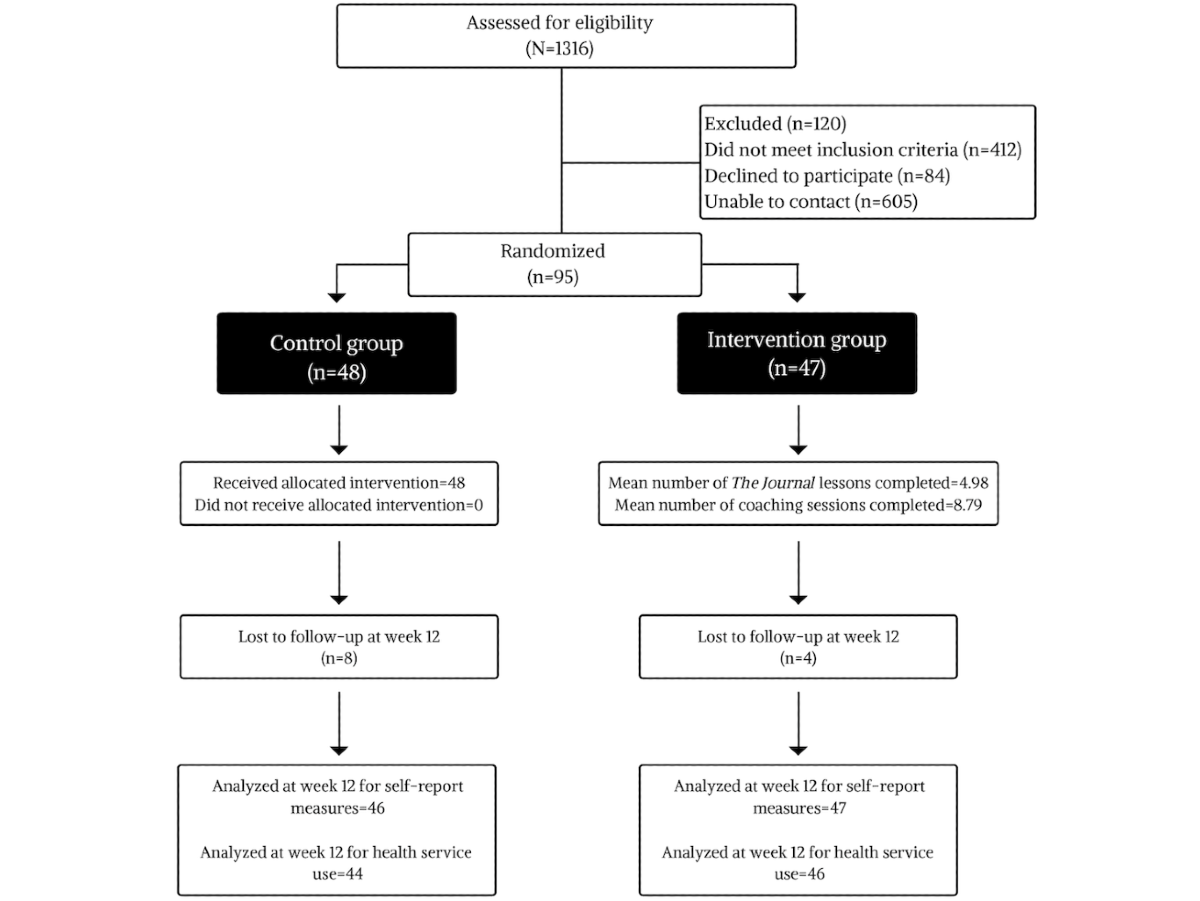
Consolidated Standards of Reporting Trials flow and attrition diagram. CONSORT: Consolidated Standards of Reporting Trials.

Recruitment for this study took place over 11 months, from May 2015 to April 2016, and a total of 1316 patients were preliminarily screened for eligibility by examining their referral documentation (Figure 1). Of these, 45.9% (605/1316) could not be reached to complete a full screening for eligibility; 40.4% (532/1316) could be contacted were not eligible to participate, and 13.6% (179/1316) were eligible and could be contacted.

Of the 45.9% (605/1316) of patients who could not be contacted to complete a full eligibility screen, 25.4% (154/605) provided consent to be approached about research studies but did not reply to telephone or mail invitations; 63.1% (382/605) were invited to discuss the study by mail with no response, and 11.3% (69/606) had incomplete mailing information listed on their referral documents.

Of the 40.4% (532/1316) of patients who could be contacted but were ineligible to participate, 84.9% (452/532) were referred for a reason other than depressive symptoms; 5.6% (30/532) had cognitive impairments rendering them unable to use a computer; 4.5% (24/532) were no longer on the waitlist at the time of screening; 2.6% (14/532) were under 16 years of age at the time of screening; 1.12% (6/532) were unable to read and write in English; and, 1.12% (6/532) did not have a valid Ontario Health Insurance Plan number.

Of the 13.6% (179/1316) of patients who were eligible to participate, 46.9% (84/179) declined to participate, and 53.1% (95/179) consented to participate in the study. Table 4 lists the reasons for patients declining to participate.

The majority of participants enrolled in the study were recruited from the Mood and Anxiety Outpatient Clinic (n=92), and 3 were recruited from the Geriatric Psychiatry Program at the ROMHC. No participants were enrolled from the Youth Psychiatry Program. Table 5 describes the demographic characteristics of the participants. There were significantly more women in the intervention group and more men in the control group (Χ^2^_1_=6.6; *P*=.01).

Participants in the intervention group completed a mean number of 5.0 (SD 2.3) lessons in *The Journal* and 8.8 (SD 3.1) sessions with the coach. In the control group, 10% (5/48) of participants reported accessing *The Journal*. In the control group, 17% (8/48) of participants scored 9 or below on the PHQ-9 at baseline compared with 23% (11/47) of participants in the intervention group. Owing to gender imbalances between the groups, we also conducted a post hoc gender analysis of changes in PHQ-9 scores and health service use.

**Table 4:** Reasons for nonparticipation (n=84).

Reason for Nonparticipation	Value, n (%)
Did not attend baseline intake appointment	27 (32)
Did not have computer/internet at home—not interested in going to public library or community center	16 (19)
No reason provided	13 (15)
Interested in participating in a different study also recruiting from the mood and anxiety program	6 (7)
Too overwhelming	4 (5)
Would prefer to wait for appointment with psychiatrist	3 (4)
Not interested in participating in research at the Royal Ottawa Hospital	3 (4)
Did not feel that the study would benefit them	2 (4)
Not interested in weekly contact	2 (2)
No time	2 (2)
Family circumstances	1 (1)
Participating in another research study	1 (1)
Interested in medication change or recommendations	1 (1)
Migraines due to computer use	1 (1)
Moving out of province	1 (1)
Interested only in face-to-face therapy	1 (1)

**Table 5:** Sample demographic characteristics.

Demographic characteristic		Total (n=95)	Control group (n=48)	Intervention group (n=47)
**Gender, n (%)^a^**				
	Male	28 (30)	20 (42)	8 (17)
	Female	66 (70)	28 (58)	38 (83)^b^
Age (years), mean (SD)		44.2 (12.9)	44.8 (13.7)	43.5 (12.1)
**Ethnicity, n (%)**				
	First Nations	1 (1)	0 (0)	1 (2)
	Inuk	0 (0)	0 (0)	0 (0)
	Métis	3 (3)	3 (6)	0 (0)
	Asian	3 (3)	2 (4)	1 (2)
	African, Caribbean, or Black	0 (0)	0 (0)	0 (0)
	White	82 (87)	42 (88)	40 (87)
	Other	5 (5)	1 (2)	4 (9)
**Marital status, n (%)**				
	Single	33 (35)	11 (23)	22 (47)
	Common law	6 (6)	4 (8)	2 (4)
	Married	35 (37)	18 (38)	17 (36)
	Separated	4 (4)	3 (6)	1 (2)
	Divorced	16 (17)	11 (23)	5 (11)
	Widowed	1 (1)	1 (2)	0 (0)
**Education level, n (%)**				
	High School	10 (11)	7 (15)	3 (6)
	College	45 (47)	23 (48)	22 (47)
	University—undergraduate	26 (27)	10 (21)	16 (34)
	University—master’s	10 (11)	5 (10)	5 (11)
	University—doctorate	4 (4)	3 (6)	1 (2)
**Employment status, n (%)**				
	Full-time	23 (24)	11 (23)	12 (26)
	Part-time	8 (8)	2 (4)	6 (13)
	Short-term disability	5 (5)	2 (4)	3 (6)
	Long-term disability	31 (33)	18 (38)	13 (28)
	Self-employed	7 (7)	3 (6)	4 (8)
	Retired	10 (11)	5 (10)	5 (11)
	Unemployed	11 (12)	7 (15)	4 (8)

^a^n=1 transgender participant removed from the analysis.

^b^*P*=.01.

### Primary Outcome

The mean PHQ-9 score was lower in the intervention group than in the control group at all study time points (Table 6). The proportions of missing values were comparable between the 2 groups at all assessment points, with the exception of week 12 (2/46 , 4% in the control group compared with 0/47, 0% in the intervention group). The Shapiro-Wilk test of normality revealed that all data were normally distributed with the exception of the control group at the week 12 study time point (W_46_=0.95; *P*=.04).

At 12 weeks, the mean PHQ-9 score in the intervention group was 11.3 (SD 6.4) and was 12.4 (SD 6.4) in the control group (t_86_=0.76; *P*=.45, 95% CI −1.9 to 3.8). To account for the non-normality in scores at week 12, mean differences (MDs) at this time were also assessed using a Mann-Whitney U test of significance, which confirmed that the difference between the two groups was not statistically significant (Mann-Whitney U statistic=862.0; Z=−0.85; *P*=.40). The mean change in PHQ-9 scores from baseline to week 12 was −3.6 (SD 6.6) in the intervention group and −3.1 (SD 6.2) in the control group, which was not statistically significant (t_91_=−0.37; *P*=.72, 95% CI −3.1 to 2.2).

Excluding the 10% (5/48) of participants in the control group who also accessed *The Journal* did not result in any statistically significant differences in mean PHQ-9 scores compared with the intervention group. The mean score at week 12 for the control group excluding those who accessed *The Journal* (n=41) was 12.4 (SD 6.2) compared with 10.7 in the intervention group (t_86_=0.76; *P*=.45, 95% CI −0.9 to 4.3).

**Table 6:** Mean and median scores on the Patient-Health Questionnaire-9 (n=95).

Study time point		Mean (SD)	Median	Missing values, n (%)	Mean difference (95% CI)	Independent samples *t* test (*df*)	*P* value
**Baseline**					**0.5 (−1.8 to 2.9)**	**0.45 (93)**	**.66**
	Control (n=48)	15.4 (5.4)	16.5	0 (0)			
	Intervention (n=47)	14.9 (6.0)	16.0	0 (0)			
**Week 2**					**1.1 (−1.3 to 3.5)**	**0.92 (89)**	**.36**
	Control (n=46)	12.6 (5.5)	11.5	2 (4)			
	Intervention (n=45)	11.5 (5.9)	12.0	2 (4)			
**Week 6**					**1.7 (−0.9 to 4.3)**	**1.29 (89)**	**.19**
	Control (n=46)	12.4 (6.2)	12.0	2 (4)			
	Intervention (n=45)	10.7 (6.1)	10.0	2 (4)			
**Week 12**					**1.0 (−1.9 to 3.8)**	**0.76 (89)**	**.45**
	Control (n=46)	12.4 (6.4)	11.5	2 (4)			
	Intervention (n=47)	11.3 (6.4)	10	0 (0)			

The repeated measures modeling found that scores on the PHQ-9 differed significantly by study time point, irrespective of group allocation (*F*_2.6,222.8_=11.59, *P*<.001). However, this relationship was not sustained once treatment group was taken into account (*F*_1,87_=1.46, *P*=.23). Similarly, modeling found that scores on the PHQ-9 were not significantly associated with gender (*F*_1,87_=0.33, *P*=.57) or the interaction between treatment group and PHQ-9 scores (*F*_1,87_=0.70; *P*=.53). However, results were significantly associated with age of participants (*F*_1,87_=5.97, *P*=.02, ŋ^2^=0.06).

### Secondary Outcomes

#### Health-Related Quality of Life

After 12 weeks of treatment, participants in the intervention group reported higher health-related quality of life, with mean index scores on the EQ-5D-3L of 0.7 (SD 0.7) for the intervention group and 0.6 (SD 0.5) for the control group (t_86_=−2.30; *P*=.02, CI 95% −0.2 to −0.1; Multimedia Appendix 3). Similarly, at 12 weeks, the mean EQ-5D-VAS score was significantly greater in the intervention group (mean 66.8, SD 18.0) than in the control group (mean 55.9, SD 19.2; t_84_=−2.73; *P*=.01).

#### Changes in Suicidality

At 12 weeks, of the 47 participants in the intervention group, 66% (31/47) reported no suicidality, 28% (13/47) reported suicidality several days in the preceding 12 weeks, 2% (1/47) reported suicidality nearly half the days, and 4% (2/47) reported suicidality nearly every day. In comparison, of the 46 participants in the control group, 69% (33/46) reported no suicidality, 17% (8/46) reported suicidality several days, 6% (3/46) reported suicidality more than half the days, and 4% (2/46) reported suicidality nearly every day (Table 7). These differences in suicidality were not statistically significant (Χ^2^_3_=2.2; *P*=.52).

**Table 7:** Mean and median scores on the Patient-Health Questionnaire -9, Question 9 (n=95).

Study time point			Control (n=48), n (%)		Intervention (n=47), n (%)		Chi-square (*df*)		*P* value
**Baseline**							**0.44 (3)**		**.93**
	Not at all	24 (50)		23 (49)					
	Several days	16 (33)		18 (38)					
	More than half the days	5 (11)		4 (9)					
	Nearly everyday	3 (6)		2 (4)					
	Total	48 (100.0)		47 (100.0)					
**Week 2**							**1.37 (3)**		**.71**
	Not at all	30 (63)		34 (72)					
	Several days	12 (25)		8 (17)					
	More than half the days	2 (4)		2 (4)					
	Nearly everyday	2 (4)		1 (2)					
	Total	46 (96)		45 (95)					
**Week 6**							**4.70 (3)**		**.20**
	Not at all	27 (56)		32 (68)					
	Several days	11 (23)		11 (23)					
	More than half the days	5 (10)		2 (4)					
	Nearly everyday	3 (6)		0 (0.0)					
	Total	46 (95)		45 (95)					
**Week 12**							**2.24 (3)**		**.52**
	Not at all	33 (69)		31 (66)					
	Several days	8 (17)		13 (28)					
	More than half the days	3 (6)		1 (2)					
	Nearly everyday	2 (4)		2 (4)					
	Total	46 (96)		47 (100.0)					

#### Hospital Service Use

Participants in both groups received similar levels of face-to-face follow-up care, with 25.0% of participants in the intervention group receiving any follow-up in the next 12 months at the hospital after their initial outpatient appointment with a psychiatrist compared with 21.3% of the control group (Χ^2^_1_=0.7; *P=*.80; Table 8). Participants in the intervention group attended a mean number of 4.8 (SD 8.5) face-to-face follow-up appointments compared with 3.6 (SD 5.6) in the control group (Mann-Whitney U statistic=255.50; Z=−0.11; *P*=.91).

**Table 8:** Hospital service use.

Health service use indicator	Control	Intervention	Tests of association	*P* value
Number of days from referral to first appointment^a^, mean (SD)	213.6 (54.6)	219.3 (57.0)	t_69_=−0.43 *P*	.67
Number of people who received outpatient follow-up by a nonpsychiatrist after initial assessment by a psychiatrist, n (%)	13/44 (30)	12/46 (26)	Χ^2^_1_=0.1	.71
Number of people who received outpatient follow-up by a psychiatrist after their initial consultation, n (%)	20/44 (42)	18/46 (38)	Χ^2^_1_=0.3	.54
Number of outpatient follow-up appointments with a psychiatrist in the year after the initial consultation^b^, mean (SD)	2.3 (3.1)	2.5 (4.6)	t_88_=−0.30	.76
Number of outpatient follow-up appointments with all disciplines in the year after initial consultation^c^, mean (SD)	3.6 (5.6)	4.8 (8.5)	M-W^d^ U=255.5, Z=−0.11	.91

^a^Control group n=34; Intervention group n=37.

^b^Control group n=34; Intervention group n=37.

^c^Control group n=34; Intervention group n=37.

^d^M-W: Mann-Whitney.

### Process Evaluation

#### Implementation

##### Reach

As reported above, only 13.4% (95/711) of potentially eligible patients (eg, those who could be contacted and were on the waitlist for mental health treatment) agreed to participate in the trial. Of particular note is that 8.9% (16/179) eligible participants declined to participate because they did not have computer or internet access at home. This is comparable with 14% of the Canadian population [33]. Similarly, among participants who were randomized to one of the trial arms, 42% (40/95) were university-educated compared with 33.3% of residents of Ottawa [34].

##### Fidelity

Analysis of trial records demonstrated that the study intervention was implemented as intended in the study protocol. Participants in the intervention group completed a mean of 5.0 (SD 2.3) lessons in *The Journal*. Similarly, throughout the course of the study, participants completed a mean of 8.8 (SD 3.1) telephone coaching sessions, ranging from a mean of 9.6 to 61.6 min in duration, and a mean of 21.6 (SD 10.8) contacts with the coach (mean 13.1 (SD 4.0) telephone calls, 2.0 (SD 2.5) emails, and mean 25.7 (SD 15.4) text messages. All fidelity measures were significantly associated with PHQ-9 scores at 12 weeks, with the exception of the types of contact with the coach (Table 9). Total lessons completed in *The Journal*, total number of coaching sessions completed, and average length of coaching calls were all inversely related to PHQ-9 scores at 12 weeks, with those having completed more lessons in *The Journal*, a higher number of sessions with the coach and longer coaching calls reporting lower levels of depression after 12 weeks of treatment (Table 9).

**Table 9:** Relationship between fidelity measures and Patient-Health Questionnaire scores at 12 weeks (n=47).

Fidelity Measures		Mean (SD)		Median		Pearson’s correlation with PHQ-9^a^ scores at week 12, (n=47)			
						*r*		*P* value	
Total lessons completed in *The Journal*		5.0 (2.3)		6.0		−0.436		.002	
Total sessions with the coach		8.8 (3.1)		10.0		−0.435		.002	
Average length of coaching calls (min)		30.8 (12.9)		27.7		−0.360		.01	
**Total contacts with coach by type**									
	Telephone		13.1 (4.0)		13.0		0.061		.68
	Email		2.0 (2.5)		1.0		−0.163		.27
	Text message		25.7(15.4)		26.0		−0.073		.62

^a^PHQ-9: Patient Health Questionnaire.

##### Dose

For those participants in the intervention group who received a full *dose* of the study intervention, completing 6 or more lessons from *The Journal* (n=29), the mean PHQ-9 scores at 12 weeks was 9.5 (SD 5.7) compared with 14.2 (SD 6.6) among those who completed 5 lessons or less in *The Journal* and 12.4 (SD 6.4) in the control group (*F*_2,85_=3.5; *P*=.04). Post hoc comparisons using the Tukey Honestly Significant Difference test indicated that the MD among those who completed at least 6 lessons in *The Journal* was significantly different from those who completed 5 or fewer lessons in *The Journal* (MD=−4.7, *P*=.04). However, the MDs between the control group and both experimental groups were not statistically significant (MD_≥6 Lessons_=−2.8; *P*=.15; MD_≤5 Lessons=_1.86; *P*=.55). Similarly, those who completed at least six or more lessons in *The Journal* were more likely to demonstrate a clinically significant reduction in symptoms, with 65.5% reporting a significant reduction in symptoms compared with 27.8% of those who completed less than 5 or fewer lessons and 37.0% of the control group (Χ^2^_2_=8.2; *P*=.02; Φ=0.30).

Similarly, by week 12 in the intervention group, 51% (24/47) of participants had a clinically important reduction in depressive symptoms (PHQ-9 score of 9 or less or a 50% or more improvement in scores) compared with 37% (17/46) of participants in the control group. However, this difference was not statistically significant (Χ^2^_1_=1.8; *P*=.17). Among participants in the intervention group who completed the full *dose* of the intervention by finishing 6 or more *Journal* sessions, 66% (19/29) had clinically important reductions in depression at 12 weeks compared with 37% (17/46) in the control group (Χ^2^_1_^=^6.3; *P*=.01; Φ=0.37).

#### Context and Mechanisms of Impact

A total of 15 qualitative interviews were conducted (10 intervention, 5 control) with 4 male (2 intervention group, 2 control group), 10 female (7 intervention group, 3 control group), and 1 transgender person (intervention group). Interviews took place at the ROMHC at a time that was mutually convenient for both participants and interviewers. Themes that arose from the interviewers were categorized in terms of whether there were positive facilitators of or negative barriers to engagement with the study intervention.

#### Facilitators to Engagement With The Journal

Participants identified Sir John Kirwan’s relatability as a positive facilitator through his sharing of his personal experience with depression. For instance, a participant commented on the videos that begin each lesson in *The Journal*, explaining the following:

…I just like the way that they interacted with each other and how casual it was, so it didn’t feel like I was doing homework, and it didn’t feel like I was doing medical stuff…It was just, watching two people sitting on a bench in a park and they were talking, and I really liked how the narrator came right out and said, you know, when I was depressed, you know, I couldn’t get out of bed, or I, I didn’t want to take a shower, it was, like, wow somebody else feels the way I do. So, it helped me to feel like I wasn’t all, all alone. And he was, he seemed to be very honest about his experience.

P040

Similarly, participants expressed an appreciation for the structure and layout of *The Journal*, specifically commenting on the usefulness of the Problem-Solving modules:

Interviewer: …what did you like about the problem-solving lesson?

Participant: … it was in a new, new approaches. It gave me a new technique or I found a new technique in there in terms of outlining problems, and thinking about them and, that I hadn’t.

P095

#### Barriers to Engagement With The Journal

Participants also reported frustrations with some aspects of *The Journal*, specifically relating to the technical issues they encountered. At the time of the trial, *The Journal* was not available on mobile devices, such as smartphones or tablets, which may have limited participants’ ability to access it. Similarly, technical issues presented themselves within the web-based program as well. For instance, in each lesson, participants must set deadlines for tasks that they are required to complete and are unable to move forward in *The Journal* until these deadlines pass. Participants also identified issues with motivation and questioned whether *The Journal* would be most appropriate for patients who are only mildly depressed. For instance, one participant explained:

Participant: Yeah, lifestyle one…was little bit of a challenge.

Interviewer: Yeah? In what way?

Participant: … just trying to find motivation…it’s just hard to change some things about lifestyle, but it came with time”

P002

Similarly, a participant in the control group commented on how some depressive symptoms, such as difficulty with concentration, may act as barriers to engagement with treatment:

Interviewer: … Were there any barriers or challenges to using the brochure?

Participant: …not really because it’s short and sweet…my only barrier was time, you know, time and, and energy. Because at the time, and it was almost an opportune time that I was doing this because I was low at the time… so when you are depressed the last thing you want to do it read, so you can’t even. And that’s why I was saying that if this was little bullet chunks, you know, you don’t have to read it so much, cause I can’t read a book when I’m down. Like I start to read a paragraph and I’m like, no I have to close it.

P014

#### Coach as a Mechanism of Change

Numerous participants in the intervention group also identified the coach as key to their success with the program, highlighting the importance of accountability:

Interviewer: …what would you say made the biggest difference to your participation in the study? ...was it the journal, was it the coach?

Participant: It was the coach, oh my god. I would not have stayed with that journal unless [SL] was calling me… cause then I wouldn’t really have anybody to be like, hey did you do that thing?

P038

Similarly, another participant questioned whether they would have been as successful in completing the program had they not had someone following-up on their goals:

…if it was by myself, I might have just kept postponing it or not doing it or, so I found it quite helpful. She's a pretty great coach.

P068

Similarly, another participant highlighted the importance of continuity between the lessons in *The Journal* and the content of the coaching sessions:

I like the conversations the following week with the homework that we did. I liked that it was continuous.

P069

Accountability also emerged as important for participants in the control group. For instance, 1 participant who accessed *The Journal* expressed a lack of follow-up by a third party as a barrier to completing the program, suggesting that:

…you guys can follow-up and see if they’ re.. If people are using it or not…just to say, somebody is following me, I should probably do a little bit more of this...

P066

This was echoed by another participant in the control group who struggled with the limited follow-up they received from the research team:

taking not even five minutes to say “how are you doing right now?”… So in a way it gauges their, their level of health, of mental health at the time, and if they do appear to need resources then [the Research Assistant] could say “look I’m not a doctor, I’m not a psychiatrist, but do you remember the flyer that I gave you, on it there was this thing that might be helpful for you right now, you might want to give it a thought”… so, that’s just like extra, like value added for resourcefulness.

P014

## Discussion

### Principal Findings

Among patients on a waiting list referred to secondary care for depressive symptoms, a trial of usual care plus coach-facilitated web-based therapy compared with usual care plus information-only waitlist control group found no statistically significant differences in mean depression scores after 12 weeks. However, participants who completed 6 or more lessons in *The Journal* reported significantly greater reductions in symptoms compared with controls. Participants in the intervention group also reported better quality of life after 12 weeks of treatment. There was no difference in service use between the two groups. During the qualitative interviews conducted as part of the process evaluation, participants identified the role of the coach as a major factor in their completion of the web-based therapy program.

### Strengths and Limitations

The major strength of this study is that it is the first study of coach-assisted web-based therapy in a secondary care setting in Canada. This study demonstrated that it is possible to incorporate the use of a coach within this clinical setting. However, due to limitations imposed by institutional policies, study staff were restricted in their ability to recruit participants, with only 12% (95/784) of potentially eligible patients agreeing to participate in the trial. It is reasonable to expect greater reach if this intervention was rolled out by a clinical service that could approach people directly at the time of referral.

There was also a possibility of contamination between the arms of the trial. Given that *The Journal* is a publicly accessible website, any participant was able to access it, and there was little that could be done by the study team to prevent this. However, our experience is that patients in secondary care do not widely use *The Journal*, with only 10% (5/48) participants in the control group reporting use of *The Journal* during the treatment period. Furthermore, contamination is likely to bias the study to showing no difference (as the control group could use *The Journal* unguided), so any differences that are found are likely to be more *believable*. Finally, the ability to generalize study findings to the larger Canadian population may be limited as a result of our highly educated population.

Some participants who were interviewed as part of the process evaluation expressed issues with recall given that the interviews took place 6 months after study completion. It is possible that interviews conducted closer to study completion would have yielded richer qualitative data.

### Comparison With Prior Work

A key contribution of this study is that participant engagement with and adherence to the intervention was much higher than that reported in previous work, with 62% (29/47) of participants in the intervention group receiving a full *dose* of treatment, completing an average of 5 out of 6 lessons in *The Journal* and 8.8 telephone coaching sessions with the coach. Comparatively, in the REEACT trial, participants only completed a median of 2 sessions of web-based therapy. Similarly, in the study by Kenter et al [21], only 36% of participants received the required 4 to 5 sessions of internet-based psychotherapy. This was found in spite of difficulties with ease of access, as *The Journal* was not available on mobile or tablet devices at the time of the study. This trial also highlights the importance of the uptake of the intervention to clinical outcomes, with participants who completed at least 6 lessons in *The Journal* experiencing a significantly greater reduction in depression symptoms than those who did not.

The previous trial of a guided version of *The Journal* in New Zealand secondary care showed no differences in clinical outcomes or service use. However, the patients in the New Zealand study had higher baseline depression scores, a mean PHQ-9 of 17.0, and were less engaged with the study intervention, completing only a mean of 2.5 lessons in *The Journal*. The magnitude of change in PHQ-9 scores was also different, with an intervention group change in PHQ-9 scores of 3.5 in Canada compared with 9.4 in New Zealand. This presumably reflects differences in usual care, with participants in New Zealand receiving mental health care, whereas Canadian participants received care from their primary care physicians while on a waiting list.

### Clinical Implications

Web-based therapies are often promoted as a way to address long waiting lists for mental health care services. However, in this study, even though participants in the intervention group with more exposure to the study intervention were more likely to experience a significant reduction in symptoms, this had little impact on subsequent service use. Web-based therapies are part of a complex socio-technological system and, as such, cannot exist in a vacuum. To achieve improvement in patient outcomes, they must be integrated into a larger system of care. This could be achieved with a more explicit stepped care system supported by web-based therapies, with a more flexible response from providers based on patient need.

### Unanswered Questions and Future Research

This study provides limited support for the potential use of web-based therapies within a stepped-care approach to the treatment of depression. However, an RCT is needed to determine the effectiveness of such an approach. The impact of providing digital services to those in greatest need, who are also the least likely to have access to high-speed connections, also needs to be taken into account. Future research on internet-based psychotherapy for depression needs to include the system of care in which it is used. This can be done through implementation science tools, which not only evaluate the effectiveness of web-based therapies but also the factors central to their uptake, such as reach, adoption, and sustainability.

### Conclusions

The results of this study demonstrated that the use of guided web-based therapy for the treatment of depression is not more effective than information-only waitlist control. However, it showed that coach-guided web-based therapy has the potential to increase adherence and engagement with depression treatment protocols. Greater adherence resulted in greater effectiveness. More research is needed on the human component of coaching in conjunction with web-based therapy to examine what factors lead to greater adherence. Researchers also need to consider when and how web-based therapies should be integrated into existing clinical pathways.

## Appendix

Multimedia Appendix 1 CONSORT EHEALTH Checklist V1.6.

Multimedia Appendix 2 Supplemental file – Screenshots from The Journal.

Multimedia Appendix 3 Mean and median scores on the EuroQol 5 dimension (3 levels) visual analog scale (n=94).

## Abbreviations

CBT: Cognitive behavioral therapy
EMR: electronic medical record
EQ-5D-3L: EuroQol 5 Dimensions (3 levels)
EQ-5D-VAS: EuroQol 5 dimensions visual analogue scale
MD: mean difference
NICE: National Institute for Health and Care Excellence
PHQ-9 Q9: Patient Health Questionnaire question 9
PHQ-9: Patient Health Questionnaire
RCT: randomized controlled trial.
REEACT: Randomised Evaluation of the Effectiveness and Acceptability of Computerised Therapy
ROMHC: Royal Ottawa Mental Health Centre.
