# Supplementary material for: Coach-Facilitated Web-Based Therapy Compared With Information About Web-Based Resources in Patients Referred to Secondary Mental Health Care for Depression: Randomized Controlled Trial
Source: J Med Internet Res. 2020 Jun 9;22(6):e15001. doi: 10.2196/15001 (PMC7312263; doi:10.2196/15001)
Supplement: Multimedia Appendix 2 [file jmir_v22i6e15001_app2.docx]

## Supplemental File


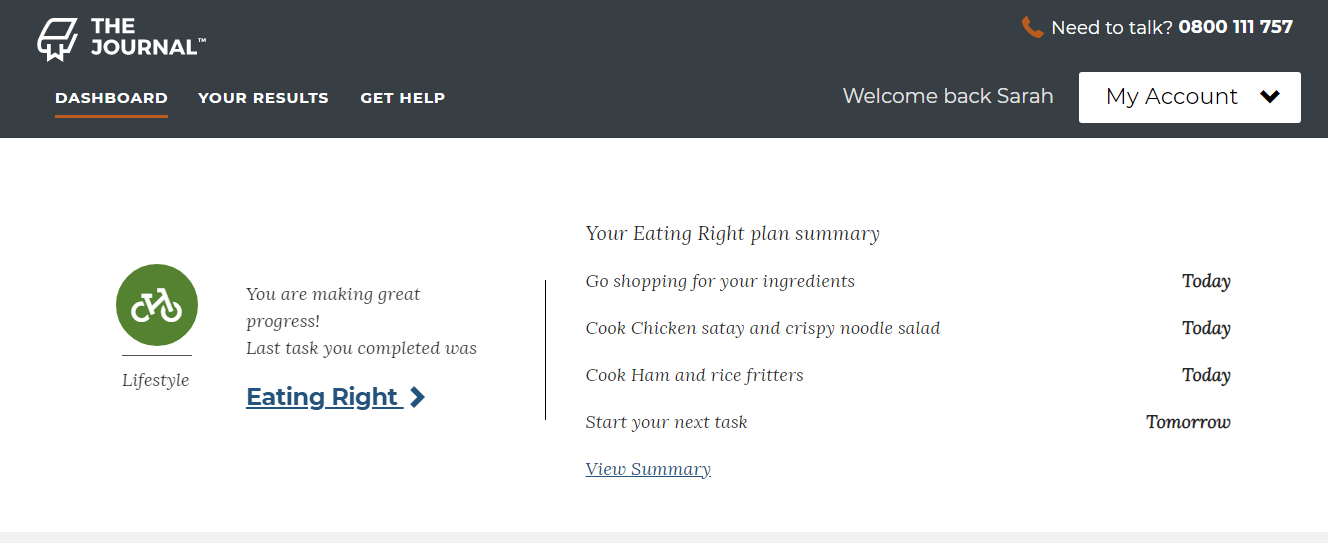


Figure 1. *The Journal* Dashboard Landing Page

^
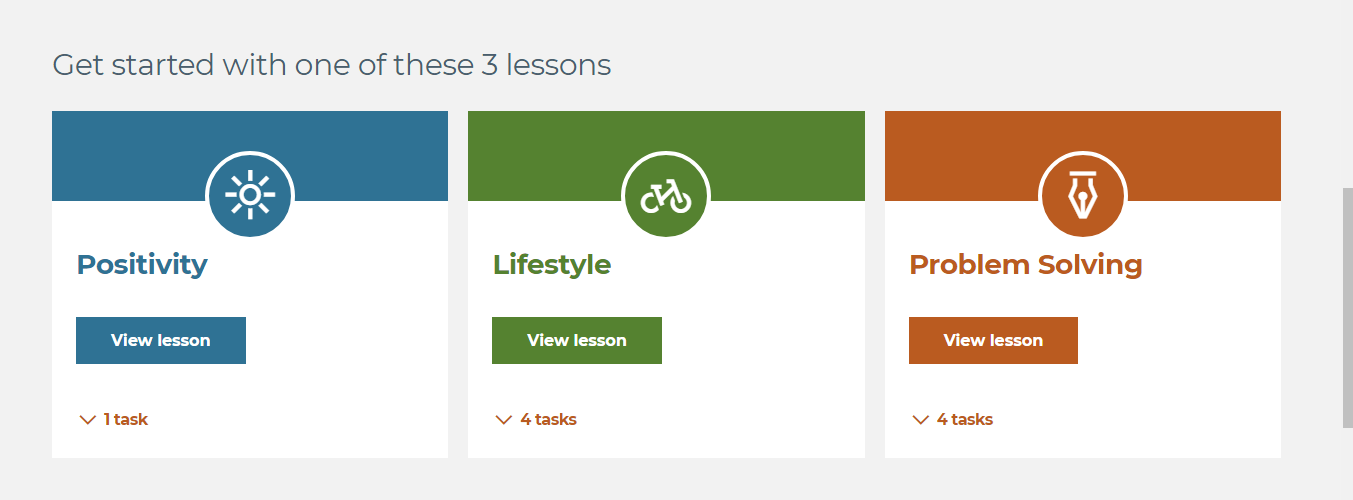
^

Figure 2. *The Journal* Lessons Overview


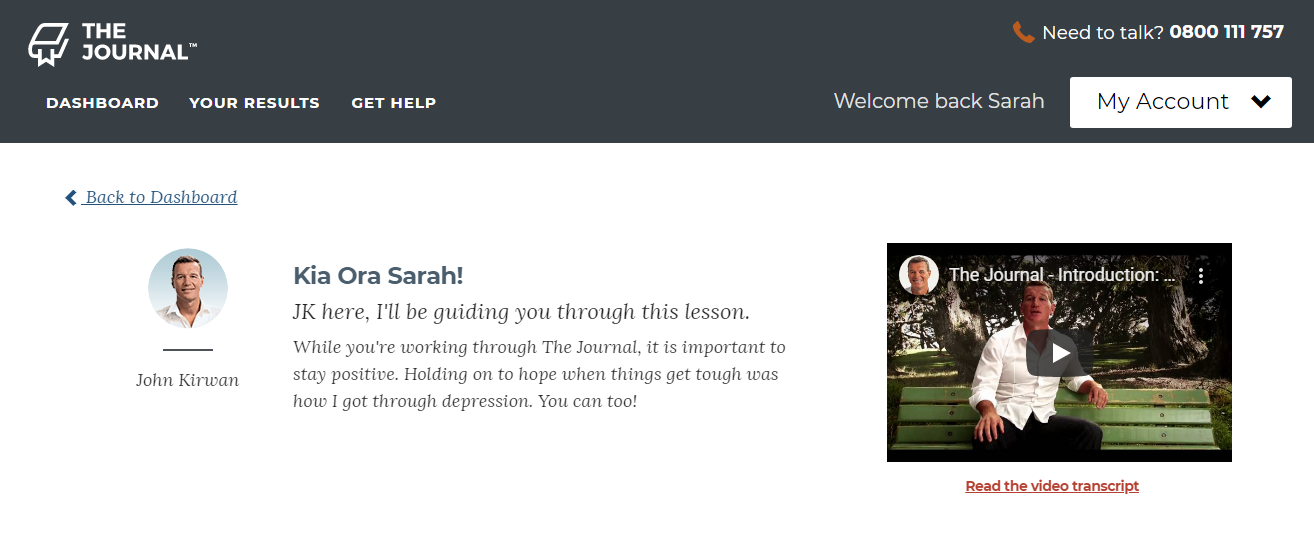


^
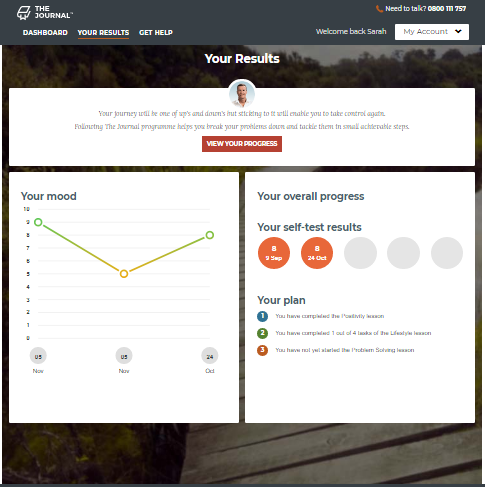
^Figure 3. *The Journal* Positivity Lesson Overview

Figure 4. *The Journal* Progress Overview
