# Supplementary material for: Coach-Facilitated Web-Based Therapy Compared With Information About Web-Based Resources in Patients Referred to Secondary Mental Health Care for Depression: Randomized Controlled Trial
Source: J Med Internet Res. 2020 Jun 9;22(6):e15001. doi: 10.2196/15001 (PMC7312263; doi:10.2196/15001)
Supplement: Multimedia Appendix 3 [file jmir_v22i6e15001_app3.docx]

|  | | Mean (SD) | Median | Missing values (%) | Mean difference  (95% CI) | Independent samples t-test | *P* value |
| --- | --- | --- | --- | --- | --- | --- | --- |
| **Baseline** | | | | | −6.6 (−14.7 to 1.4) | *t*_92_=1.63 | .11 |
|  | Control (n=47) | 51.5 (21.7) | 59.0 | 1 (2.1)^a^ |  |  |  |
|  | Intervention (n=47) | 58.2 (17.4) | 60.0 | 0 (0) |  |  |  |
| **Week 6** | | | | | −3.8 (−11.6 to 4.0) | *t*_82_=0.96 | .34 |
|  | Control (n=42) | 57.5 (19.4) | 60.0 | 6 (12.5) |  |  |  |
|  | Intervention (n=42) | 61.3 (16.4) | 64.0 | 5 (10.6) |  |  |  |
| **Week 12** | | | | | −10.9 (−18.9 to −3.0) | *t*_84_=−2.73 | .01 |
|  | Control (n=41) | 55.9 (19.2) | 60.0 | 7 (14.6) |  |  |  |
|  | Intervention (n=45) | 66.8 (18.0) | 70.0 | 2 (4.3) |  |  |  |

^a^n=1 excluded from the analysis for refusal.
